# Supplementary material for: A fault-intrusion-tolerant system and deadline-aware algorithm for scheduling scientific workflow in the cloud
Source: PeerJ Comput Sci. 2021 Nov 2;7:e747. doi: 10.7717/peerj-cs.747 (PMC8576554; doi:10.7717/peerj-cs.747)
Supplement: Supplemental Information 6 [file peerj-cs-07-747-s006.zip › Myproject1.1/docs/org/cloudbus/cloudsim/provisioners/package-summary.html]

org.cloudbus.cloudsim.provisioners


JavaScript is disabled on your browser.


- Overview
- Package
- Class
- Use
- Tree
- Deprecated
- Index
- Help

- Prev Package
- Next Package

- Frames
- No Frames

- All Classes

# Package org.cloudbus.cloudsim.provisioners

- Class Summary

  | Class | Description |
  | --- | --- |
  |  |  |
  | --- | --- |
  | BwProvisioner | BwProvisioner is an abstract class that represents the provisioning policy of bandwidth to virtual machines inside a Host. |
  | BwProvisionerSimple | BwProvisionerSimple is a class that implements a simple best effort allocation policy: if there is bw available to request, it allocates; otherwise, it fails. |
  | PeProvisioner | The Class PeProvisioner. |
  | PeProvisionerSimple | The Class PeProvisionerSimple. |
  | RamProvisioner | RamProvisioner is an abstract class that represents the provisioning policy of memory to virtual machines inside a Host. |
  | RamProvisionerSimple | RamProvisionerSimple is an extension of RamProvisioner which uses a best-effort policy to allocate memory to a VM. |

- Overview
- Package
- Class
- Use
- Tree
- Deprecated
- Index
- Help

- Prev Package
- Next Package

- Frames
- No Frames

- All Classes
